# Metal levels of canned fish sold in Türkiye: health risk assessment

**DOI:** 10.3389/fnut.2023.1255857

**Published:** 2023-10-31

**Authors:** Ali Riza Kosker, Sedat Gundogdu, Tuba Esatbeyoglu, Deniz Ayas, Fatih Ozogul

**Affiliations:** ^1^Department of Seafood Processing Technology, Faculty of Fisheries, Cukurova University, Adana, Türkiye; ^2^Department of Basic Science, Faculty of Fisheries, Cukurova University, Adana, Türkiye; ^3^Department of Food Development and Food Quality, Institute of Food Science and Human Nutrition, Gottfried Wilhelm Leibniz University Hannover, Hannover, Germany; ^4^Fisheries Faculty, Mersin University, Mersin, Türkiye; ^5^Biotechnology Research and Application Center, Cukurova University, Adana, Türkiye

**Keywords:** tuna fish, salmon, mackerel, toxic metals, Inductively Coupled Plasma–Mass Spectrometer

## Abstract

This study analyzed 34 canned fish products, including 28 tuna specimens, 3 salmon specimens, 1 mackerel specimen, and 1 anchovy specimen, from 13 different brands purchased in Türkiye. The study aimed to determine metal/metalloid levels in canned fish and potential health risks for both children and adult consumers. The metal/metalloid levels in the samples were determined using an Inductively Coupled Plasma–Mass Spectrometer (ICP–MS), with the range of levels found as follows (mg/kg, ww): Fe (12.12–101.4), Cu (2.19–11.68), Zn (4.06–33.56), Se (0.24–10.74), Al (1.41–14.45), Cr (0.06–4.08), Pb (0.10–0.43), Cd (0.001–0.110), and As (0.01–0.13). Estimated weekly intake (EWI) levels were found that the consumption of canned fish products did not pose any risk based on the EWI levels and provisional tolerable weekly intake (PTWI) limits. However, three tuna samples had target hazard quotient (THQ) levels above the threshold (>1). Arsenic levels were found to increase the carcinogenic risk for child consumers if they heavily consumed 18 canned fish products, including 15 tuna, 2 salmon, and 1 mackerel. The maximum allowable consumption rates (CRmm) for each canned fish product were calculated monthly. Consequently, the consumption of canned fish by children can pose health risks.

## 1. Introduction

Canning is a healthy alternative food to reduce the occurrence of non-communicable diseases caused by nourishment (1). This process was established after close observation of heat treatment and high-quality preservation of food stored in sealed glass bottles. It became even more common right after the invention of metal cans. Canned fish was first introduced to the United States in 1815. Fish such as tuna, shad, and alewives were first canned in the early twentieth century (2). With the COVID-19 pandemic, consumers have tended to pay more attention to their diet, and the place of fish in the diet and canned products has increased due to consumer demand. In this period, processed and packaged products were preferred more than fresh or chilled products all over the world (3–9). Processed product consumption in Europe increased from 424 thousand tons to 511 thousand tons from 2019 to 2020 with the pandemic (10). The increase in consumers' preference for processed and packaged products instead of fresh or chilled products has increased the purchase of canned products. For instance, canned food consumption increased 21% in Portugal, 14% in Italy, and 13% in Luxembourg during the pandemic period (11). The increase in consumers' orientation toward canned products caused an increase of 7% in all tuna fish imports in Europe and 11% in filet tuna imports in 2020 (10). However, as with all food products, there are risks in canned products that may adversely affect consumer health. For example, in canned fish, which is one of the most common canned products, contaminations can be observed in the transportation and processing processes, as well as the contaminations that may occur in the habitat of the fish (12–15). For this reason, it is essential to monitor regularly the canned products offered to the consumer for contaminants. In recent years, studies on microplastic and Bisphenol-A contamination in canned products have also been carried out (15–19). There is also a lot of research on metal pollution, one of the most common risks in canned fish (13, 14, 20–32). Studies on metal contamination in food products have largely concentrated on the quantification aspect. Authorities such as the European Union (EC, 1881/2006), World Health Organization (WHO), the US Environmental Protection Agency (US EPA), and the Turkish Food Codex (TGK) have already established limit values with regard to the potential risks of metal contamination of food products for human consumption. However, estimating the health risks that the metal levels in the product will create for the consumers has become even more essential regarding food safety. As a result, the evaluation of metal contamination in food products has expanded to include both consumer health and ecological pollution (38). In addition to establishing limit values, several consumer risk assessment criteria, such as estimated weekly intake (EWI), target hazard quotient (THQ), and lifetime cancer risk (CR), have become increasingly relevant to ensure consumer health and food safety. Moreover, contamination of processed seafood can also occur during transportation, processing and packaging (12, 14, 24). For this reason, it is important for food safety to calculate the metal levels and related health risk estimation calculations of the canned seafood products offered to consumers, which have an increasing market share worldwide.

Therefore, this study investigated health risk calculations based on metal/metalloid levels in 34 canned fish samples of 13 different brands purchased from grocery stores in Turkey during the summer of 2021. Initially, the levels of elements such as Fe, Cu, Zn, and Se were investigated due to their effects on the nutritional quality of canned fish products and potentially toxic metals/metalloids such as Al, Cr, Pb, Cd, and As, which have a risk of adversely affecting consumer health. Then, based on the metal/metaloids concentrations, health risk calculations were carried out. In this context, the calculations of health risk analysis (EWI, THQ, and CR) were made in adults and children in case of consumption once, three, and 5 days a week to predict the possible risks in terms of consumer health consuming of 34 different canned fish products. In addition, the maximum allowable consumption for canned fish was measured daily and monthly for the samples examined.

## 2. Materials and methods

### 2.1. Canned fish

This study involved the procurement of 34 canned fish samples from seven companies that were obtained from local markets in Türkiye in 2021. These canned fish samples were derived from various fish species such as Black Sea anchovy, Norwegian salmon, longtail tuna, yellowfin tuna, skipjack, and mackerel (Table 1).

**Table 1:** Characteristics of sampled canned fish. In the code column, the letters indicate brands, and the numbers indicate products.

**Code**	**Package type**	**Fish species**	**Additives**	**Product weight**	**Party-no**
P-1	Can	Tuna	Water, salt	160 g	9283
P-2	Can	Tuna	Sunflower oil (%27), water, salt	80 g	20842E
D1	Can (BPA free)	Yellowfin Tuna	Olive oil, salt	75 g	19/10/2020
D2	Can	Skipjack	Sunflower oil, canola oil, salt	80 g	07-09-2020
D3	Can (BPA free)	Yellowfin Tuna	Water	75 g	05-10-2020
D4	Aluminum	Norwegian Salmon	Olive oil, salt	100 g	16.11.2024
D5	Aluminum	Skipjack	Olive oil, salt	125 g	10-06-2023
D6	Aluminum	Blacksea Anchovy	Sunflower oil, salt	110 g	23.09.2024
D7	Aluminum	Mackerel	Olive oil, salt	110 g	19-10-2024
D8	C/PP (90)	Norwegian Salmon	Olive oil, lemon water, salt	85 g	27.01.2022
D9	C/PP (90)	Skipjack	Sunflower oil, salt	120 g	20.10.2022
D10	C/PP (90)	Yellowfin Tuna	Water	120 g	18-03-2023
D11	C/PP (90)	Yellowfin Tuna	Olive oil, salt	185 g	02-06-2023
D12	Glass	Skipjack	Olive oil, salt	185 g	25.09.2023
C1	Can	Tuna	Sunflower oil, salt	80 g	20848E
W1	Can	Tuna	Sunflower oil, salt	160 g	20861E CO
DE1	Can	Tuna	Sunflower oil, salt	160 g	20861E
MI1	Can	Tuna	Sunflower oil, canola oil, salt	160 g	20860E CO
M1	Can	Tuna	Water, salt	80 g	25
M2	Can	Skipjack	Sunflower oil (%25), water, salt	80 g	26
M3	Can	Yellowfin Tuna	Olive oil (%25), water, salt	160 g	46
Y1	Can	Tuna	Sunflower oil (%25), water, salt	104 g	20
Y2	Aluminum	Mackerel	Sunflower oil, water, salt	160 g	14
SF1	Can	Tuna	Olive oil (%25), water, salt	75 g	0265
SF2	Can	Tuna	Sunflower oil (%27), water, salt	80 g	0051
SF3	Can	Tuna	Sunflower oil, water, salt	80 g	0197
SF4	Can	Tuna	Water, salt	80 g	7339
V1	Can	Tuna	Sunflower oil, water, salt	160 g	0346
F1	Can	Tuna	Sunflower oil, salt	160 g	L1720
SA1	Can	Salmon	Sunflower oil, water, salt	160 g	302507280
SA2	Can	Tuna	Water, salt	160 g	435375812
SA3	Can	Tuna	Olive oil, salt	160 gr	3021078T3
SA4	Can	Tuna	Sunflower oil, salt, water	80 gr	406009812
T1	Can	Tuna	Sunflower oil, salt	80 gr	17848E

### 2.2. Elemental analyses

Metal analysis of canned fish was performed using the method of Canli and Atli (33). Canned fish samples with a wet weight (ww) of 0.1 g were treated using a solution of 2 ml perchloric acid and 4 ml concentrated nitric acid (Merck, Darmstadt, Germany). The canned fish samples were subjected to digestion by placing them on a hot plate set at 150°C until complete dissolution of the tissue. The levels of various trace elements including Iron (Fe), zinc (Zn), copper (Cu), selenium (Se), aluminum (Al), cadmium (Cd), chrome (Cr), lead (Pb), and arsenic (As) present in the canned samples (mg/kg) were determined using Inductively Coupled Plasma-Mass Spectrometer (ICP-MS) (Agilent, 7500ce, Japan). The operating conditions of ICP-MS were as below: radio frequency (RF) 1,500 W; plasma gas flow rate, 15 L min^−1^; auxiliary gas flow rate, 1 L min^−1^; carrying gas flow rate, 1.1 L min^−1^; spray chamber T, 2°C; sample depth, 8.6 mm; sample entry rate, 1 mL min^−1^; nebuliser pump, 0.1 rps. The ICP-MS was calibrated with a high-purity multi-standard (Charleston, SC 29423) mixture for the elemental analysis. Standard solutions for calibration curves were prepared by the dilution of a stock solution of selected elements. Standard solutions prepared for toxic metals were in the 1–50 ppb (0.001–0.050 mg/L) range, while for macro and trace elements, they were in the 1–50 ppm (1–50 mg/L) range. The accuracy of the metal analysis was ensured through the use of the International Atomic Energy Agency's (IAEA) Certified Reference Material (CRM) IAEA-436. The IAEA reference material prepared by the International Atomic Energy Agency's Marine Environmental Studies Laboratory (MESL) was used for tuna meat homogenate. The same methodology as that used for analyzing the samples under study was employed for the reference material utilized in this research. The certified value of the IAEA436 reference material was compared to the observed value. Repeated analysis of the reference material demonstrated good accuracy (Table 2). The limit of detection (LOD) for Fe, Cu, Zn, Se, Al, Cr, Cd, and Pb and As, were 0.018, 0.056, 0.108, 0.027, 0.001, 0.007, 0.0004, and 0.048 and 0.003, mg/kg, respectively (Table 2).

**Table 2:** The confirmed and observed values of reference material (IAEA-436) and the quantitative limits for the elements.

**Analyte**	**Certificated value (mg/kg)**	**Observed value (mg/kg)**	**95% Confidence interval (mg/kg)**	**Recovery %**	**LOD (mg/kg)**	**LOQ (mg/kg)**
Fe	88	86 ± 4.30	80.0–92.0	97.73	0.0175	0.0541
Cu	1.74	1.73 ± 0.04	1.68–1.79	99.31	0.0565	0.1985
Zn	18.00	17.2 ± 1.30	16.00–19.00	95.56	0.1075	0.3165
Se	4.43	4.25 ± 0.25	3.97–4.51	95.98	0.0275	0.0846
Al	3.92	3.83 ± 0.07	3.76–3.92	97.76	0.001	0.0031
Cr	0.13	0.13 ± 0.01	0.11–0.14	98.46	0.0068	0.0229
Cd	0.05	0.05 ± 0.00	0.04–0.05	96.87	0.0004	0.0012
Pb	-	0.10 ± 0.00	-	-	0.0379	0.0965
As	1.98	1.96 ± 0.04	1.91–2.02	98.89	0.0026	0.0086

LOD, Limit of detection; LOD, Limit of quantification.

### 2.3. Health risk estimation

In order to assess the risks associated with consuming the canned fish samples, EWI, THQ, and CR values were calculated for consumption frequencies of once, three, and five times per week. In health risk estimation calculations, using seafood consumption data (16.82 g/person/day) provided by Turkish Statistical Institute (T.S.I., 2020) since specific canned fish consumption data for Turkey from the were not available. The calculations were conducted separately for both adults and children. According to data from the United States Environmental Protection Agency (34), a body weight of 70 kg and a lifespan of 70 years for adult consumers, and a body weight of 32 kg for children (35) and a lifespan of seven years were considered.

All metals except for As were directly analyzed using instrumental analysis values. Total As has a higher proportion of organic forms than inorganic forms, with organic As being less toxic than the inorganic form (36). Consequently, this makes it difficult to assess the potential health risks associated with its concentration in fish samples (37, 38). To evaluate the risk factors (EWI, THQ, and CR) associated with As concentration, the toxic form was assumed to be 3% of the total As concentration, as suggested in previous studies (38–41).

EWI was calculated using the formula determined by USEPA (34):


(1) $$\begin{array}{l} EWI=\left(C_{M}.CR\right)/BW \end{array}$$

The EWI equation used in this study includes the metal concentration (CM), consumption rate (CR), and consumer body weight (BW). The calculated EWI values were compared with the provisional tolerable weekly intake (PTWI) levels established by the FAO/WHO Joint Expert Committee on Food Additives (JECFA) and the European Food Safety Authority (EFSA). PTWI represents the lifetime weekly intake of a substance in food or drinking water that is unlikely to cause significant health risks, based on body weight (mg/kg body weight).

The THQ calculation represents the ratio of exposure to metals, metalloids and reference doses (RfD), which is used to assess the non-carcinogenic risks of metals. THQ values were determined using methods established by the USEPA (42).


(2) $$\begin{array}{l} THQ=\left[\left(EF.ED.CR.C_{M}\right)/\left(RfD.BW.AT\right)\right].10^{-3} \end{array}$$

In this equation, EF represents the frequency of exposure to the metal or metalloid of interest at 52, 156, and 260 days per year for weekly, 3 and 5-day exposures, respectively. ED stands for lifetime exposure time. This is 70 years for adults while it is 7 years for children. CR represents the consumption rate, and CM represents the metal concentration in the tissues of the samples investigated. RfD represents the oral reference dose. Based on US EPA (42) data, RfD values used for As, Cd, Pb, Cr, Fe, Cu, Zn, Se, and Al are 3.10^−4^, 1.10^−3^, 4.10^−3^, 3.10^−3^,0.7, 0.04, 0.3, 5.10^−3^, and 1.00, respectively. BW indicates body weight. As reported by USEPA, 70 kg was used for adults and 32 kg for children. AT indicates the average non-carcinogenic time; the AT value was calculated as 365 days/year × ED. The THQ value of >1 indicates that consuming the examined canned fish samples may cause different non-carcinogenic health problems for consumers (42, 43).

∑THQ is the sum of the THQ values of all elements studied.


(3) $$\sum^{\text{​}}THQ(TTHQ)=THQ_{As}+THQ_{Al}+\ldots +THQn)$$

CR calculations were applied according to US EPA, 2019. CR calculates cancer risk in people exposed to metal pollution through consumption. CR values above 10^−5^ include a high risk of developing cancer.


(4) $$\begin{array}{l} CR=\left[\left(EF.ED.CR.C_{M}.CsF\right)/\left(BW.AT\right)\right].10^{-3} \end{array}$$

The CR equation includes a modification where the cancer slope factor (CsF) value is used. For the metals Pb, As, Cr, and Cd, the *CsF* values used were 8.5.10^−3^, 1.5, 0.5, and 6.3, respectively, according to the US EPA (42).

### 2.4. Maximum allowable consumption rate

The US EPA suggests that the daily limits on fish consumption should be expressed as the number of meals that can be safely consumed in a given period for a specific meal size. Therefore, in this study, the daily fish consumption limit (CRlim) and the number of meals per month (CRmm) were calculated. For non-carcinogenic heavy metals, the CRlim was determined using Equation (5). For carcinogenic metals and metalloids (Cd, Cr, Pb, and As), CRlim was calculated using Equation (6).


(5) $$\begin{array}{l} CRlim:\left(RfD.BW\right)/C_{M} \end{array}$$

(6) $$\begin{array}{l} CRlim^{*}:\left(ARL.BW\right)/\left(C_{M}.CSF\right) \end{array}$$

Information on the number of meals that a consumer can safely consume is more practical than daily limits. The maximum allowable consumption rate, CRmm, is expressed in terms of the number of meals per month. If the consumption rate of a contaminated fish species is more than 16 meals per month, it suggests that consuming this species does not pose a significant risk to human health (34). Therefore, the number of meals allowed per month for a consumer was calculated by considering multiple pollutants for both carcinogenic and non-carcinogenic effects using the following Equation (7) proposed by the US EPA (34):


(7) $$\begin{array}{l} CRmm:\left(.CR\text{lim.}T\text{ap}\right)/\text{M}S \end{array}$$

In these Equations (5–7), ARL is the maximum acceptable individual lifetime risk level (unitless; it was used the risk level of 10–5). The TAP refer to the average time interval (365.25 days/12 months = 30.44 days/month) and MS refer to the amount of food per meal that is 0.227 kg fish/meal for adults, 0.114 kg fish/meal for children.

### 2.5. Statistical analyses

Results are reported as the mean and standard deviation of the measurements. SPSS version 17.0 (SPSS Inc., Chicago, IL. USA) was used for statistical evaluations of the changes of values among 34 different brands for each metals. To determine significant differences between the levels detected for each metal/metalloid in canned fish samples, a one-way analysis of variance (ANOVA) combined with Duncan's multiple range test comparisons at *p* < 0.05 were performed.

## 3. Results and discussions

Fe, Cu, Zn, Se, Al, Cr, Cd, Pb, and As levels (mg/kg, ww) were determined for 34 canned fish samples from 13 brands with three replicates purchased from Turkish markets during the summer of 2021 (Table 3). In addition, consumer health risk assessment was performed by calculating EWI and THQ values (Table 3), as well as CR and CRmm values (**Tables 5**, **6**, respectively).

**Table 3:** Metal levels in 34 canned fish products (mg/kg ww).

**Products**	**FeMean ±SD**	**CuMean ±SD**	**Zn Mean ±SD**	**SeMean ±SD**	**Al Mean ±SD**	**Cr Mean ±SD**	**Pb Mean ±SD**	**Cd Mean ±SD**	**As Mean ±SD**	**Fish species**
P-1	70.78 ± 10.1	10.3 ± 5.16	15.6 ± 0.87	5.89 ± 0.34	4.67 ± 0.57	1.81 ± 0.48	0.43 ± 0.01	0.001 ± 0.00	0.05 ± 0.00	Tuna
P-2	29.54 ± 2.06	11.7 ± 0.65	9.07 ± 0.72	5.88 ± 0.65	3.52 ± 0.45 0.45	0.36 ± 0.08	0.26 ± 0.03	0.001 ± 0.00	0.07 ± 0.00	Tuna
D1	101.4 ± 3.28	5.47 ± 0.25	17.0 ± 0.51	8.34 ± 0.29	NA	0.99 ± 0.06	0.34 ± 0.03	0.002 ± 0.00	0.08 ± 0.00	Yellowfin Tuna
D2	22.77 ± 0.57	4.88 ± 0.41	6.86 ± 1.08	4.57 ± 0.83	3.88 ± 0.29	4.08 ± 0.67	0.27 ± 0.03	0.001 ± 0.00	0.04 ± 0.00	Skipjack
D3	49.51 ± 2.30	3.23 ± 0.15	9.28 ± 0.43	1.29 ± 0.06	14.45 ± 0.67	0.61 ± 0.03	0.31 ± 0.01	0.001 ± 0.00	0.04 ± 0.00	Yellowfin Tuna
D4	15.90 ± 0.89	2.19 ± 0.21	8.70 ± 0.44	2.76 ± 0.10	3.13 ± 0.49	0.12 ± 0.05	0.27 ± 0.04	0.013 ± 0.00	0.07 ± 0.01	Norwegian Salmon
D5	40.61 ± 4.42	2.47 ± 0.26	7.21 ± 0.57	3.38 ± 0.33	8.56 ± 0.54	0.10 ± 0.10	0.27 ± 0.03	0.014 ± 0.00	0.07 ± 0.00	Skipjack
D6	44.25 ± 3.12	4.60 ± 0.81	33.6 ± 3.69	0.23 ± 0.03	7.89 ± 0.60	0.23 ± 0.23	0.34 ± 0.02	0.012 ± 0.00	0.02 ± 0.00	Anchovy
D7	13.16 ± 1.24	4.80 ± 0.88	5.85 ± 0.53	3.38 ± 0.33	6.44 ± 0.48	0.50 ± 0.14	0.26 ± 0.03	0.003 ± 0.00	0.13 ± 0.00	Mackerel
D8	13.31 ± 0.31	3.69 ± 0.22	11.0 ± 0.83	1.43 ± 0.21	6.08 ± 0.49	0.25 ± 0.05	0.10 ± 0.02	0.011 ± 0.00	0.03 ± 0.00	Norwegian Salmon
D9	26.76 ± 1.82	3.88 ± 0.28	13.7 ± 0.80	2.35 ± 0.36	3.71 ± 0.50	0.20 ± 0.06	0.23 ± 0.03	NA	0.06 ± 0.00	Skipjack
D10	20.79 ± 1.29	3.73 ± 0.71	14.6 ± 0.65	3.79 ± 0.59	5.17 ± 0.86	0.15 ± 0.03	0.17 ± 0.03	0.003 ± 0.00	NA	Yellowfin Tuna
D11	17.56 ± 1.42	2.63 ± 0.26	9.33 ± 0.95	5.94 ± 0.65	8.35 ± 0.44	0.27 ± 0.03	0.18 ± 0.02	0.027 ± 0.00	NA	Yellowfin Tuna
D12	25.38 ± 2.66	3.38 ± 0.28	10.6 ± 0.31	1.13 ± 0.78	13.8 ± 1.18	0.16 ± 0.07	0.17 ± 0.00	0.110 ± 0.01	0.04 ± 0.01	Skipjack
C1	41.39 ± 2.40	3.40 ± 0.65	12.6 ± 0.98	4.69 ± 0.35	3.85 ± 0.74	0.13 ± 0.01	0.22 ± 0.05	0.009 ± 0.00	0.06 ± 0.01	Tuna
W1	86.88 ± 10.0	3.62 ± 0.40	13.3 ± 0.66	6.69 ± 1.62	13.8 ± 1.42	0.18 ± 0.06	0.27 ± 0.03	0.109 ± 0.03	0.10 ± 0.00	Tuna
DE1	32.97 ± 2.20	3.13 ± 0.27	6.29 ± 0.64	5.13 ± 0.57	5.24 ± 1.17	0.06 ± 0.01	0.25 ± 0.08	0.108 ± 0.02	0.12 ± 0.01	Tuna
MI1	27.13 ± 1.66	2.92 ± 0.21	11.6 ± 1.85	3.53 ± 0.09	4.46 ± 0.36	0.11 ± 0.02	0.15 ± 0.05	0.099 ± 0.00	0.02 ± 0.00	Tuna
M1	59.84 ± 3.67	6.73 ± 0.38	15.0 ± 1.63	2.67 ± 0.98	8.04 ± 0.51	0.24 ± 0.09	0.23 ± 0.01	0.001 ± 0.00	0.05 ± 0.00	Tuna
M2	16.70 ± 0.57	2.35 ± 0.21	8.60 ± 0.91	1.93 ± 0.18	3.63 ± 0.65	0.17 ± 0.02	0.33 ± 0.07	0.047 ± 0.01	0.05 ± 0.00	Skipjack
M3	12.81 ± 1.18	2.86 ± 0.26	8.16 ± 0.75	1.91 ± 0.16	2.68 ± 0.25	0.12 ± 0.02	0.18 ± 0.02	0.018 ± 0.00	0.04 ± 0.00	Yellowfin Tuna
Y1	42.70 ± 1.82	2.57 ± 0.10	7.60 ± 0.49	4.84 ± 0.60	6.38 ± 0.31	0.09 ± 0.06	0.16 ± 0.00	NA	0.03 ± 0.00	Tuna
Y2	97.36 ± 2.67	2.90 ± 0.22	11.4 ± 2.37	2.98 ± 0.72	13.5 ± 1.76	0.31 ± 0.02	0.27 ± 0.06	0.040 ± 0.00	0.09 ± 0.01	Mackerel
SF1	35.47 ± 2.34	8.60 ± 0.57	4.06 ± 0.27	4.29 ± 0.28	1.98 ± 0.13	0.31 ± 0.02	NA	0.001 ± 0.00	0.05 ± 0.00	Tuna
SF2	12.12 ± 1.09	6.05 ± 0.50	5.31 ± 0.71	10.7 ± 1.26	NA	NA	0.23 ± 0.00	0.001 ± 0.00	0.02 ± 0.00	Tuna
SF3	30.67 ± 2.50	4.08 ± 0.72	15.7 ± 1.97	2.78 ± 0.37	5.63 ± 0.91	0.18 ± 0.04	0.41 ± 0.05	0.010 ± 0.00	0.02 ± 0.00	Tuna
SF4	35.17 ± 20.5	2.53 ± 0.47	10.9 ± 0.92	4.62 ± 0.78	4.13 ± 0.47	0.23 ± 0.11	0.25 ± 0.05	0.010 ± 0.00	0.02 ± 0.00	Tuna
V1	22.17 ± 1.71	4.15 ± 0.81	24.6 ± 1.66	7.37 ± 0.61	7.82 ± 0.97	0.48 ± 0.36	0.26 ± 0.02	0.002 ± 0.00	0.01 ± 0.00	Tuna
F1	37.69 ± 4.61	2.58 ± 0.23	8.52 ± 0.94	4.61 ± 0.14	11.5 ± 3.97	0.12 ± 0.01	0.29 ± 0.06	0.032 ± 0.00	NA	Tuna
SAS1	14.04 ± 2.13	5.09 ± 0.59	8.35 ± 7.13	4.43 ± 2.23	1.41 ± 0.15	0.17 ± 0.04	0.21 ± 0.06	0.001 ± 0.00	0.04 ± 0.00	Salmon
SAS2	30.14 ± 3.37	7.63 ± 0.31	15.2 ± 1.30	5.09 ± 0.63	2.03 ± 0.26	0.32 ± 0.18	0.20 ± 0.02	0.002 ± 0.00	0.03 ± 0.00	Tuna
SAS3	32.91 ± 1.50	6.54 ± 0.35	12.3 ± 0.59	1.66 ± 0.59	10.7 ± 1.70	0.19 ± 0.10	0.27 ± 0.01	0.003 ± 0.00	0.07 ± 0.01	Tuna
SAS4	31.50 ± 3.03	4.53 ± 0.48	14.3 ± 1.37	8.09 ± 0.76	6.01 ± 0.48	0.12 ± 0.01	0.21 ± 0.03	0.056 ± 0.00	0.07 ± 0.01	Tuna
T1	40.93 ± 2.08	2.21 ± 0.26	5.74 ± 0.53	2.93 ± 0.16	14.1 ± 0.31	0.28 ± 0.07	0.16 ± 0.04	0.003 ± 0.00	0.06 ± 0.01	Tuna

NA, Not analyzed.

### 3.1. Metal/metalloid levels of canned fish samples

**Figure 1:**
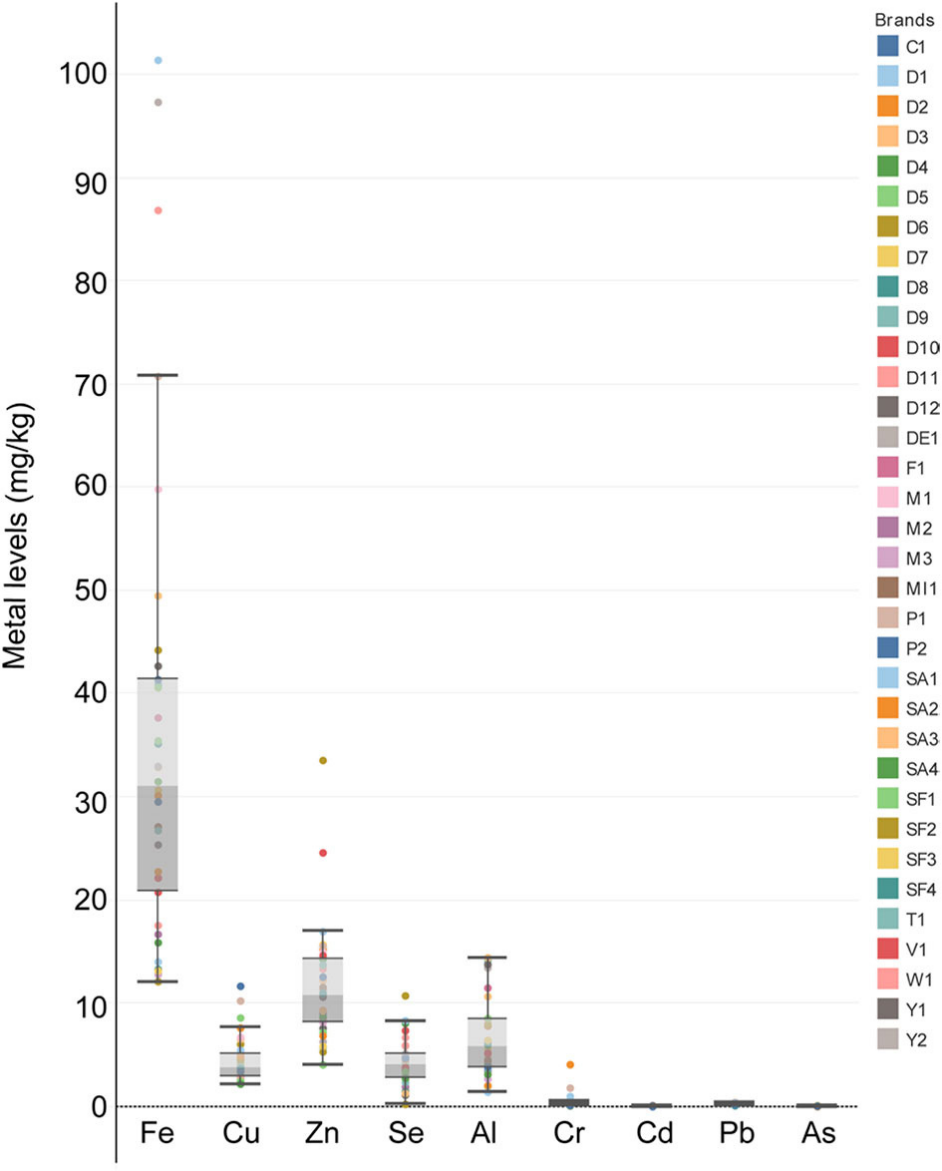
Metal levels in 34 canned fish products.

The levels of Fe, Cu, Zn, Se, Al, Cr, Cd, Pb, and As in different canned fish samples are presented in Table 3. Iron, Cu, Zn, and Cr values were observed in all samples (Figure 1). However, in a few samples, Al, Cd, and As concentrations were <LOD values. The highest metal levels were found for Fe, Zn, and Al in canned fish samples while the lowest amounts were found for Cr, Cd, Pb, and As. The mean of metal/metalloid concentrations found in all canned fish samples was as follows (mg/kg, ww): Fe (36.25), Cu (4.45), Zn (11.52), Se (4.16), Al (6.77), Cr (0.41), Cd (0.02), Pb (0.25), and As (0.05). Mean metal/metalloid concentrations were compared with maximum limit values set by the Turkish Food Codex (TFC), Food and Agriculture Organization (FAO)/World Health Organization (WHO) and European Commission (EC). FAO/WHO (44) maximum limits for Cu, Zn, Cd, and As were reported as 30, 40, 0.5, and 0.3 mg/kg, respectively. The EC (45) and TFC (46) reported maximum limits for Cd and Pb as 0.05 and 0.2 and 0.05 and 0.3 mg/kg, respectively. The mean Cu concentration is above the maximum limits reported by FAO, while the Zn concentration is below the maximum limit. Cd value were below the maximum limits for all codexes. While the mean Pb concentration was below the maximum limit value according to FAO and TFC, it was above the EC limits. In terms of statistical evaluation, significant differences were observed between the samples for Al. There are statistical differences between D3 and all other samples (*p* < 0.05). For Cr, there are statistical differences between P1, D1, and D2, and there are statistical differences between these three and all others (*p* < 0.05). For Fe, there was no statistical difference between Y2 and D1 (*p* > 0.05), while a statistical difference was found between these two and all other samples (*p* < 0.05). For Cu, while there were statistical differences between SF1, SAS2, and P2, there were also statistical differences between these three and all other samples (*p* < 0.05). For Zn, there were statistical differences between V1 and D6, while these two were statistically different from all other samples (*p* < 0.05). For Se, statistical differences were found between SF2 and all samples (*p* < 0.05). For As, there was a statistical difference between W1 and D7, while these two samples were statistically different from all other samples (*p* < 0.05). For Pb, there was no statistical difference between SF3 and P1 (*p* > 0.05), while statistical differences were found between these two and all other groups (*p* < 0.05). For Cd, while there was no statistical difference between D12 and MI1 (*p* > 0.05), these two were statistically different from all other groups (*p* < 0.05).

Iron, Cu, Zn, and Se are essential for fish nutrition (26). In this study, Fe, Cu, Zn, and Se values in canned fish are similar to the values reported by the several authors (13, 14, 22, 26, 29). It was determined that the highest Fe, Zn, and Se values were found in tuna fish compared to the canned fish species tested. Similarly, Alcala-Orozco et al. (14) found that these elements were higher in tuna whereas it was determined that Cu levels were high in tuna and anchovy. Tuzen and Soylak (21) also reported similar results. Another remarkable finding in the canned anchovy samples examined is the high levels of Zn, which is in agreement with work of Tuzen and Soylak (21) since higher zinc levels was reported by these researcher in canned anchovy. However, the Se values were lower in canned anchovy compared to other investigated canned fish samples. Selenium is a nutritionally essential trace element for the activity of over 30 enzymes with vital functions. Nevertheless, the canning process can decrease the Se level (47), which has a negative effect on the nutritional value of canned anchovies.

One of the important elements in terms of consumer health is Al. Aluminum is closely associated with many neurological diseases, such as Alzheimer's, Parkinson's, and MS (48). Although Al has been studied relatively less in canned fish, in the current study Al values observed in all four canned fish species were higher than in several studies (21, 49, 50). Al Ghoul et al. (22) reported similar levels of Al values in canned tuna. High Al values may be due to contamination in the fish's transportation, processing, and packaging processes. It is known that in canned fish products, transportation, processing, and packaging processes can cause contamination as well as the habitat of the fish used for canning fish products (14).

In this study, metals and metalloids such as Cr, Cd, Pb, and As, which may pose health risks for consumers, were also investigated. ATSDR (51) reported that Cd, Pb, and As elements are among 10 most dangerous toxic substances in the Priority List of Hazardous Substances, while Cr metal is among the 100 most dangerous substances (38). The canned fish product with the highest Cr and Cd levels was tuna. Mackerel was the species of canned fish with the highest value for As (0.11 mg/kg), a critical toxic metalloid. Chromium, Cd, Pb, and As values in tuna samples were determined as 0.43, 0.03, 0.25, and 0.05 mg/kg, respectively. The level of Cr, Pb, and Cd values found in this study are similar to the values by Alcala-Orozco et al. (14), Miedico et al. (20), Kowalska et al. (23), Ulusoy (24), Ashraf et al. (25), Novakov et al. (26), Popovic et al. (27), and Rahman et al. (29). Arsenic values in canned fish, in contrast, are similar to the values by Ulusoy (24); however, it is lower than reported by several studies (14, 26, 29, 49). This difference is due to the direct use of As values determined as a result of instrumental analyses in most studies. However, a significant portion of the As value in fish is organic, and organoarsenic are not as toxic as inorganic forms (36). Therefore, the toxic As value is assumed to be 3% of the total As concentration (37–41).

Girolametti et al. (52) reported Cd, Pb, and Fe levels in wild and farmed tuna fish as 0.01 and 0.02, 0.11 and 0.03, 13 and 7, respectively. Although the reported Cd levels were similar to the present study, Pb and Fe levels were lower than the present study. This difference may be due to the additives such as oil and water used during the canning process and may also be related to the size of the fish used in packaging. For example, in the study by Milatou et al. (53) investigating the metal levels of Atlantic bluefin tuna fish according to different size groups; Fe levels in tuna fish of 250–289 cm length are similar to the data in the present study, while there are differences in other lengths. This emphasizes how crucial it is to provide comprehensive information on packaging regarding the methods of processing and the fish utilized. Because there is a possibility that product quality may be affected during transportation and canning processes.

### 3.2. Health risk analysis

EWI, THQ (Table 4), and CR (Table 5) values were calculated to assess consumer health risks associated with the consumption of canned fish samples from the different brands. The present calculations were based on the assumption that individuals in two age groups (children and adults) consumed canned fish at different frequencies, including once, three times, or five times a week.

**Table 4:** Estimated weekly intake (EWI; μg/kg BW) and target hazard quotient (THQ) values for each metal analyzed according to different consumption frequencies.

	**Consumer groups**	**Day**	**Fe**	**Cu**	**Zn**	**Se**	**Al**	**Cr**	**Cd**	**Pb**	**As**
**EWI**	A	5	44.86	5.49	14.36	5.14	8.44	0.51	0.03	3.04	0.06
		3	26.91	3.30	8.61	3.08	5.06	0.30	0.02	1.82	0.04
		1	8.97	1.10	2.87	1.03	1.69	0.10	0.01	0.61	0.01
	C	5	98.13	12.02	31.41	11.24	18.46	1.11	0.07	6.65	0.13
		3	58.88	7.21	18.85	6.74	11.08	0.66	0.04	3.99	0.08
		1	19.63	2.40	6.28	2.25	3.69	0.22	0.01	1.33	0.03
**THQ**	A	5	0.01	0.02	0.01	0.15	0.00	0.24	0.00	0.11	0.03
		3	0.01	0.01	0.00	0.09	0.00	0.14	0.00	0.06	0.02
		1	0.00	0.00	0.00	0.03	0.00	0.05	0.00	0.02	0.01
	C	5	0.02	0.04	0.01	0.32	0.00	0.53	0.01	0.24	0.06
		3	0.01	0.03	0.01	0.19	0.00	0.32	0.01	0.14	0.04
		1	0.00	0.01	0.00	0.06	0.00	0.11	0.00	0.05	0.37

A, adult; C, children.

Exposure time of 5. 3 and 1 days/week. Arsenic calculation was made by assuming the inorganic.

**Table 5:** Target carcinogenic risk (CR) values for Cr, As, Cd, and Pb according to different consumption frequencies.

	**Consumer groups**	**Day**	**Cr**	**Cd**	**Pb**	**As**
		5	**3.5E-05**	**2.89E-05**	3.57E-06	**1.31E-05**
	A	3	**2.1E-05**	**1.74E-05**	2.14E-06	7.84E-06
CR		1	7.01E-06	5.78E-06	7.15E-07	2.61E-06
			5	**7.66E-05**	**6.33E-05**	7.82E-06	**1.31E-04**
	C	3	**4.6E-05**	**3.79E-05**	4.69E-06	**1.72E-05**
		1	**1.53E-05**	**1.27E-05**	1.56E-06	5.72E-06

A, adult; C, children.

Exposure time of 5. 3 and 1 days/week. CR values >10^−5^ are indicated in bold.

#### 3.2.1. EWI

The results of the study indicated that the estimated weekly intake (EWI) values of all the metals detected in canned fish were lower than the provisional tolerable weekly intake (PTWI) limits set by the relevant authorities (Table 4). The PTWI levels for iron (Fe), copper (Cu), and zinc (Zn) were determined by the Food and Agriculture Organization/World Health Organization (FAO/WHO) in 1983, while the levels for aluminum (Al) were established by the Joint Expert Committee on Food Additives (JECFA) in 2011. The European Food Safety Authority (EFSA) set the PTWI level for arsenic (As) in 2009. The PTWI levels for Fe, Cu, Zn, Al, and As were established at 5,600, 125 μg/kg/day, a range of 300–1,000, 2,000, and 15 μg/kg/day, respectively. Since there is no PTWI value for Se determined by the authorities, a PTWI calculation was not carried out. The tolerable monthly intake of cadmium (Cd) was updated to 25 μg/kg body weight (bw) by the Joint FAO/WHO Expert Committee on Food Additives (JECFA) in 2013. However, in this study, the provisional tolerable weekly intake (PTMI) was converted to PTWI, and a weekly value of 6.25 μg/kg bw was used instead. The ratios of EWI to PTWI for Fe, Cu, Zn, Al, Cd, and As ranged from 0.05 to 4.88%, 0.43 to 25.19%, 0.01 to 9.05%, 0.02 to 1.95%, and 0.01 to 2.33%, respectively. The calculations showed that the EWI levels were higher in children than in adults, as expected. Additionally, an increase in the frequency of canned fish consumption led to higher EWI levels. Based on the EWI levels and PTWI limits, the consumption of the canned fish samples was deemed safe with regards to the studied metals. Besides, some studies report that the EDI/EWI values in canned fish are acceptable (29, 31, 32). Rahman et al. (29) reported acceptable EDI values for As, Cr, Cd, Cu, and Zn. Herrera-Herrera et al. (32) reported that Cd EDI/EWI values in canned tuna samples were similar to results of the current study, but the Zn value was higher. Ulusoy (24) reported slightly higher EDI/EWI values in research with 222 different canned fish than the present study. There are few studies on EDI/EWI values in canned fish. Additionally, most of these studies reported that EDI/EWI values for Fe, Cu, Zn, Se, Al, Cr, Cd, and As are under PTDI/PTWI levels determined by regulatory authorities (24, 32). EWI was calculated for Pb although no PTWI comparison was made. Even though there was established PTWI for Pb (25 μg/kg bw), the FAO/WHO, based on analysis of epidemiological data, noted that the Pb of PTWI provided was associated with an increase in systolic blood pressure in adults and at least 3 points IQ loss and adverse neurodevelopmental effects in children. For this reason, it was reported that the PTWI value for Pb could not be considered (Table 5) protective for health and was therefore withdrawn, and a new PTWI that could be regarded as protective for health could not be formulated (54).

#### 3.2.2. Target hazard quotient

Based on the THQ calculations, it was found that the values for Fe, Cu, Zn, Se, Al, Cr, Cd, and As were below the threshold value of 1, as shown in Table 4. The THQ value is not a direct measure of the health risks associated with exposure to metal or metalloid pollutants, but rather serves as an indicator of potential risk (38). A THQ value >1 indicates that the amount of metal intake exceeds the RfD, as defined by the US EPA (42) and Yi et al. (43), which suggests that the metal poses a risk to the consumer. The order of metals based on THQ in both adult and child age groups is Cr > As > Se > Pb > Cu > Cd > Fe > Zn. The mean THQ values of all 34 canned fish samples for adult and child consumers were <1. In singular samples, THQ levels above the threshold value were calculated for Cr only in three samples (P1, D2, and D2). The concentrations of all other metals and metalloids were found to be below the threshold value of 1, as presented in Table 4. Similarly, Ulusoy (24) for Cd and As; Mansouri et al. (31) for Cd; Rahmani et al. (50) for As, Se, Cu, Al, Zn, and Fe reported THQ values below the threshold value (=1). Among all THQ values calculated according to the metal levels found in all canned fish samples, the highest values were observed for chromium (Cr).

Due to the Cr levels in canned fish, one of the examined samples (P1) (tuna) had non-carcinogenic risks when consumed 5 days a week for adults and 3 and 5 days a week for children. While consumption of canned yellowfin Indian ocean tuna fish (D1) did not pose any risk for adults, it is risky for children to consume it 3 days a week. In the canned Pacific skipjack tuna sample (D2), however, according to the THQ values calculated for Cr, consumption by children was risky under all conditions and for adults more than once a week. The fact that this situation was observed in a small number of tuna samples suggested that there might be contamination during processing. Salmon, mackerel, and anchovy samples examined were not THQ risky for adults and children.

TTHQ, which indicates the cumulative non-carcinogenic risk associated with exposure to all studied elements, was also evaluated in this study. A TTHQ value > 10 suggests that there may be non-carcinogenic risks that could cause health problems for consumers over an extended period. However, the results of this study indicate that not all tested canned fish samples pose a risk for TTHQ to both adults and children.

#### 3.2.3. Lifetime cancer risk

The mean CR values for Cd and Cr of all 34 canned fish samples were found to be risky in children under all conditions and adults when consumed 3 and 5 days a week (Table 5). According to the US EPA (34), the probability of a healthy individual developing cancer is 10^−5^. Therefore, the CR value is expected to be below this threshold. Values for CR > 10^−5^ include a high risk of developing cancer. For arsenic, intensive consumption was found to be risky. Mansouri et al. (31) reported that CR values in relation to Cd levels were similar to the current study when four different cans of tuna were consumed once a week. While four other products pose carcinogenic risks in relation to chromium levels when consumed heavily, the risk was determined in only one sample (P1) for adults if consumed 5 days a week. In this study, no risk was determined for adult consumers regarding carcinogenic risk owing to arsenic levels. Nevertheless, it was determined that carcinogenic risk increased for child consumers if 18 different products, including 15 tuna, two salmon, and a can of mackerel were consumed 5 days a week. Ulusoy (24) found 222 samples of canned tuna from 36 countries to have high CR values depending on the amount of As consumed 3 days a week or more. Rahmani et al. (50) also stated that attention should be paid to As of CR values originating from canned fish consumption. Arsenic, a naturally occurring metalloid, is widely distributed and considered to be the most significant toxic substance in terms of potential harm to human health due to its known or suspected toxicity. It is known to be a potent poison, a co-carcinogen, and even at low concentrations, has been shown to cause damage to almost all major organs, including the lungs, liver, brain, and bladder (55). Therefore, regular monitoring of toxic metals in processed seafood, especially As levels is important for consumer health. The CR values for Pb in 34 canned fish samples did not show any carcinogenic risk in children and adults.

### 3.3. Maximum allowable limits

**Figure 2:**
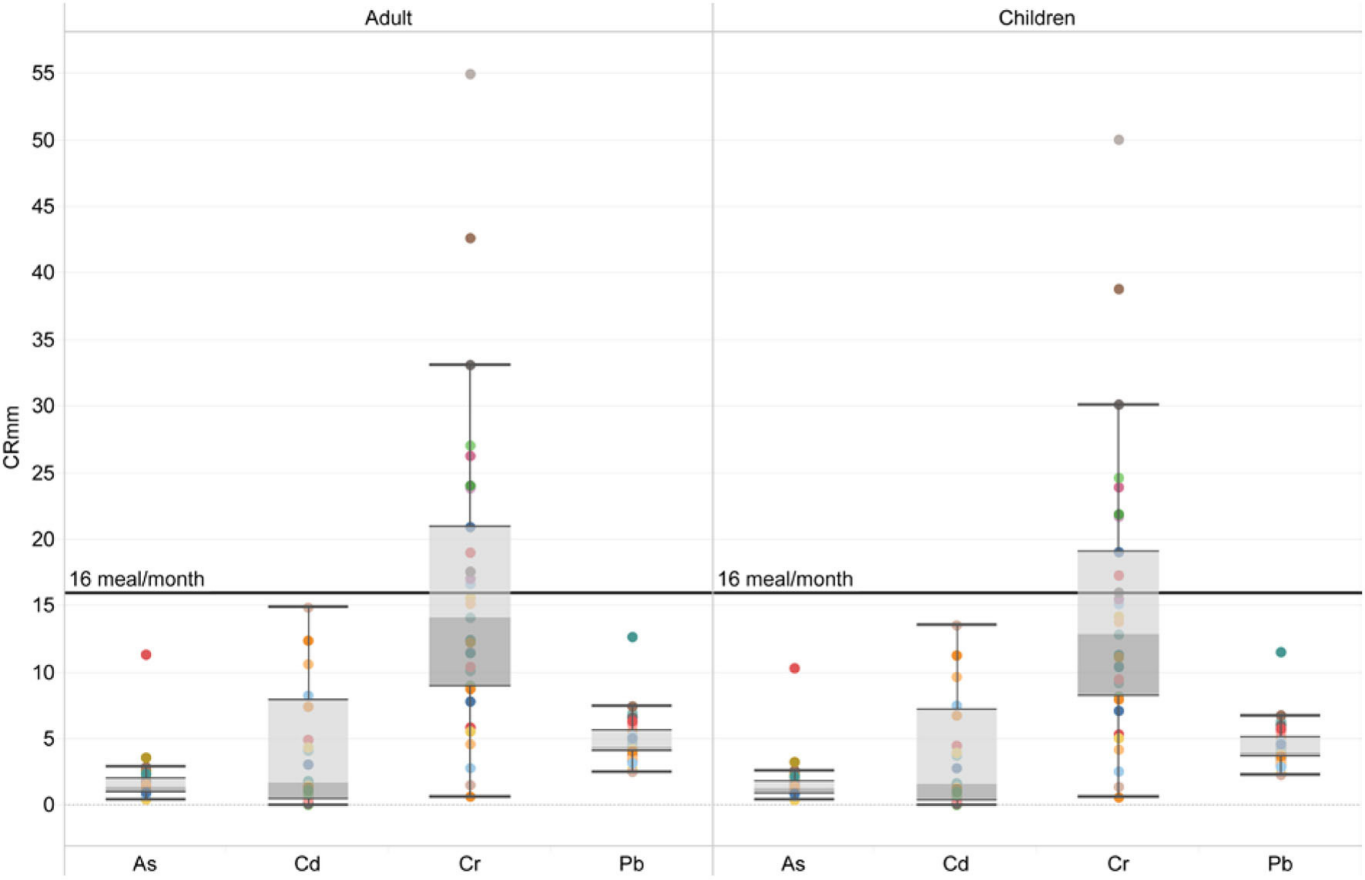
The maximum allowable consumption rate (CRmm) for Cr, As, Cd, and Pb.

The US EPA recommends the maximum allowable consumption rate (CRmm) for daily fish consumption limits to express the permissible number of fish meals consumed in a given meal size and a given period. If the number of meals of a contaminated fish species is <16 per month, it is thought that consuming this fish species may pose a risk to human health (34). In this study, CRmm values for Fe, Cu, and Zn were <16 (meals/month) for both children and adults (Table 6). While the risk for chromium was not detected in adults, it was found to be 14.68 (meals/month) in children. However, monthly food consumption levels were low for both consumer groups for Cd, Pb, and As. Particularly As levels are quite limiting. Health risks can be observed if it is consumed more than 1.70 meals per month for children and 1.87 meals per month for adults (Figure 2).

**Table 6:** The maximum allowable consumption rates for each investigated metal.

		**Fe**	**Cu**	**Zn**	**Se**	**Al**	**Cr**	**Cd**	**Pb**	**As**
A	CR(lim)kg/day	1.86	0.76	2.16	0.16	14.70	0.12	0.04	0.04	0.01
	CR(mm)	>16	>16	>16	>16	>16	>16	4.74	4.95	1.87
C	CR(lim)kg/day	4.49	2.27	5.17	0.48	32.54	0.25	0.10	0.07	0.02
	CR(mm)	>16	>16	>16	>16	>16	13.89	3.98	4.29	1.63

>16: More than 16 meals in a month.

According to all these findings, it was determined that 34 different canned fish products tested did not contain significant risks for consumers in terms of EWI and THQ. It was determined that Fe, Cu, Zn, Se, and Al levels in canned fish were not risky regarding health risk assessment in terms of metals and metalloids tested. However, it was reported that toxic metals such as Cr, Cd, Pb, and As carried carcinogenic risks and risks in terms of CRmm. It was determined that Cr, Cd, and As levels in canned fish were risky for adult and child consumers if consumed 3 days or more a week. In addition, it was observed that toxic metals had a restrictive effect on canned fish consumption in terms of maximum permissible consumption rate (CRmm). Metals and metalloids have long been recognized as critical toxic agents causing acute and chronic poisoning cases in environmental exposure situations (56). These health risks from canned fish consumption were thought to arise from the processing process. During the process of food packaging and preservation, metals can act as a source of contamination and can contaminate food through various pathways (12). Contamination can occur during food processing owing to the direct interaction of equipment, tanks, tubes, as well as other parts of processing equipment prepared from toxic metal. Moreover, contamination can also occur throughout the entire container, especially during storage stages such as canning and packaging. Although the 34 different canned products tested contain different fish caught from different regions, similar risk values were observed, particularly concerning toxic metals.

## 4. Conclusion

Canned fish has been a popular food globally for many years because of its long shelf life and microbiological protection from the canning process. However, the fish may be contaminated by metals, pesticides, microplastics, etc., from its habitat or during processing. A study of the potential health impacts of metal content in 34 canned fish products found that higher attention should be paid to contamination from processing. Although EWI levels in tuna, salmon, mackerel, and anchovy were not found to pose a risk, increased THQ and CR values were observed with intensive consumption. Although fish consumption in Türkiye is lower than in other countries, it is still important to test regularly the canned fish for toxic metals and metalloids to protect consumer health. To ensure the safety of canned fish, there are regulations and standards in place to control its production. Regular monitoring and careful regulation of production facilities can minimize contamination and provide consumers with the confidence that the canned fish they purchase is safe for consumption.

## Data availability statement

The original contributions presented in the study are included in the article/supplementary material, further inquiries can be directed to the corresponding authors.

## Author contributions

TE: Funding acquisition, Writing—review and editing. AK: Conceptualization, Data curation, Formal analysis, Investigation, Methodology, Writing—original draft. SG: Conceptualization, Data curation, Investigation, Writing—original draft. DA: Data curation, Formal analysis, Writing—review and editing. FO: Data curation, Writing—review and editing.
